# Commentary: Physical time within human time

**DOI:** 10.3389/fpsyg.2022.1025476

**Published:** 2022-10-14

**Authors:** Kristie Miller, Danqi Wang

**Affiliations:** ^1^Department of Philosophy, School of Humanities, The University of Sydney, Darlington, WA, Australia; ^2^Department of Philosophy, King's College London, London, United Kingdom

**Keywords:** flow, persistence, endurance, time, temporal experience

## A reconciliation

Gruber et al. (2022) and Buonomano and Rovelli (2022) aim to render consistent the picture of time delivered to us by physics, with the way time seems to us in experience. Their general approach is similar; they take the picture of our world given to us in physics, a picture on which there is no global “moving” present and hence no robust temporal flow, and attempt to explain why things nevertheless seem to us as they do, given that our world is that way. In this, they follow in the footsteps of Hartle (2005), Callender (2017), and Ismael (2017), who argue that any information gathering system (an IGUS) will, in learning to navigate our world, represent the distinctions between past, present, and future, and represent their own changing trajectory through spacetime. While we are generally very sympathetic to this approach, there are several places where we disagree.

## What to reconcile?

While Gruber et al. and Buonomano and Rovelli each take themselves to be attempting to bridge the gap between two ways of thinking about our world, the gap in question is a little different. Gruber et al. take themselves to be attempting to bridge the gap between the manifest image—the image of time had by each of us in ordinary experience—and the scientific image—the image presented to us in our best science. Buonomano and Rovelli's target is a little different. They take themselves to be attempting to bridge the gap between the way neuroscientists suppose things to be, in theorizing about our how we come to represent and experience the world, or perhaps even *the way neuroscientists* suppose that things *seem to us, in ordinary experience*, and how the image of the world presented to us in the scientific image. The former is straightforwardly a claim about the manifest image. The latter is a claim about what the scientific image (neuroscience) tells us about the manifest image. In what follows we will talk directly about these claims about the manifest image.

Both Gruber et al. and Buonomano and Rovelli hold that it is part of the manifest image that time passes: that it seems to us as there is a present moment, and that which moment that is, changes. Gruber et al. express this as the claim that it seems to us as though there is a *unique*, changing present. In addition, Buonomano and Rovelli hold that it is part of the manifest image (according to neuroscience) that presentism is true: that is, that only present things exist (past and future ones do not) though *which* things are present, changes. It's worth distinguishing two different claims that might be at issue here. The first is a claim about the way the world is presented to us in experience; the way it *experientially seems* to us. The second is a claim about how we take the world to be, pretheoretically; what we tend to *believe* about the world. We think that one aspect of their target—the presentist component—is a mistake.

## Presentism

Ultimately, as we read them, Buonomano and Rovelli argue that the manifest image *as it really is*, rather than as neuroscientists suppose it to be, is consistent with the scientific image. We agree. Consider first the idea that we tend to believe that presentism is true. In fact, empirical evidence regarding people's beliefs suggests that *most* people believe that past, or past and future, objects exist (Latham et al., 2019, 2020). Most people do not have a manifest image of our world as being a presentist world. We also think that Buonomano and Rovelli are right to argue that there is little reason to think that the best description of our experiences is that it seems to us as though *only* the present exists. The fact that we are usually perceptually aware of what seems to us to be a single moment, the present, and that what we are aware of, changes, does not show that it seems to us as though there *only exist* present things, any more than the fact that typically each of us is only perceptually aware of what is spatially local to us, suggests that our experiences are such that it seems as though only things that are “around here” exist. So while neuroscientists might tend to model our experiences in terms of a single, changing, present, there is nothing in our experiences themselves that suggests that we experience the world as one in which presentism is true.

## An illusion of flow

Buonomano and Rovelli hold that our experiences are *veridical* experiences of a *local changing indexical present*. According to the block universe model we are located at multiple locations in spacetime. At different locations we have different experiences. Further, because of entropy increasing away from the low entropy big bang, there are records (such as memories) of earlier events but not later ones, so at different locations our experiences represent that at earlier locations *we had* different experiences. We represent that our experiences change. Buonomano and Rovelli conceive of this as having a veridical experience of a local changing present. In this, they agree with Ismael (2012, 2017) and Sattig (2019a,b), who hold that representing these experiences as changing constitutes our having a veridical experience of time flowing. More generally, many block theorists hold that we have veridical experiences of *anemic flow:* the kind of flow that is present in block worlds and is consistent with physics (Dainton, 2011; Deng, 2013, 2019; Hoerl, 2014; Baron and Miller, 2018; Miller, 2019; Miller et al., 2020; Leininger, 2021). These authors deny that we have experiences of *robust* flow: experiences as of there being a *unique present* that changes, and hence they deny that our experiences of flow are illusory.

By contrast, Gruber et al. argue that our cognitive systems generate an illusion as of there being a unique changing present, where this illusion is a “more satisfying experience of physical time, [that produces] better adaptive behavior.” But we see little reason to suppose that the relevant experiences here are indeed illusory.

To be illusory, our experiences would need to represent that there is a *unique present* that changes. We see little reason to think they do. Consider the way we represent things *as present*. Perhaps we perceptually represent *indexical* presentness. If so, perceptual experience is tensed: it is part of the content of perception that we represent the event perceived *as occurring at the time of the perception* (Peacocke, 1999; Kriegel, 2009; Phillips, 2014). In experiencing what is indexically present as changing, however, our experiences are veridical: what is indexically present does change in a block world. Or perhaps we do not represent *presentness* at all. Hoerl (2018), holds that things presented to us in perceptual experience are not presented to us *as* present because our perceptual experience has no temporal viewpoint. Then we are not subject to any illusion. Since we see little reason to suppose that people represent that there is a *unique global* present that changes, we doubt that they are subject to an illusion of flow: instead, they have veridical experiences of anemic flow.

## Persistence: Endurance and perdurance

A second aspect of Gruber et al.'s account that we doubt is their appeal to the role of persistence in explaining the illusion of flow. Gruber et al. hold that endurantism is incompatible with a block world, so objects perdure. But if objects perdure then they do not persist. Since we have experiences as of objects, particularly the self, persisting, then those experiences are illusory, and they contribute to the illusion of flow.

While some argue that endurantism is incompatible with eternalism (Merricks, 1994, 1999; Barker and Dowe, 2003, 2005; Effingham and Robson, 2007; Giberman, 2017; Baron and Miller, 2018) it is generally held that the two are compatible (Haslanger, 1989; Sider, 2001; Miller, 2004; Brower, 2010; Eagle, 2010; Daniels, 2014; Wasserman, 2016). So we should not conclude that if our world is a block world, then objects must perdure. Moreover, even if objects do perdure, it does not follow that our experiences of persisting things are illusory. Gruber et al. write, “…perdurantism…suggests that object persistence is not veridical (Gruber et al., 2022, p. 5).” This implies that perduring objects do not persist. However, endurantism and perdurantism are accounts of persistence: they simply disagree about the *way* in which objects persist.

If we experience persisting objects as enduring, when in fact they perdure, then our experience would be illusory. Prosser (2007, 2012, 2016) takes this to be so, and he thinks we *mistake* these illusory experiences for experiences of flow. But recent empirical research by Baron et al. (2022) tends to undermine this. Baron et al. (a) found that most non-philosophers did not judge that objects endure rather than perdure, and (b) found no association between people judging that our world contains robust flow and judging that objects endure rather than perdure and (c) found that when presented with a description of an experience of time robustly flowing, people were no more inclined to judge that the world was one containing enduring rather than perduring objects.

Perhaps when Gruber et al. talk about *enduring* as opposed to *perduring* selves they really have in mind the view that there is an *unchanging core* persisting self rather than a *series of short-lived momentary selves* that have *no unchanging properties*. Then the suggestion that it is because we experience ourselves as having an *unchanging core*, that we are subject to an illusion of flow. We take it to be an open question both whether people do experience themselves as having an unchanging core, and whether, if they do, they would mistake this as an experience of flow (as per Prosser's suggestion) or that this would partially constitute them having an illusory experience as of flow (as we take it Gruber et al. are suggesting).

While the IGUS-driven approach has much to recommend, we are not convinced by the dualistic model on which the IGUS not only has veridical experiences of a block world, but also has adaptive illusory experiences as of time flowing. We see little reason to posit this second aspect to experience.

## Author contributions

All authors listed have made a substantial, direct, and intellectual contribution to the work and approved it for publication.

## Conflict of interest

The authors declare that the research was conducted in the absence of any commercial or financial relationships that could be construed as a potential conflict of interest.

## Publisher's note

All claims expressed in this article are solely those of the authors and do not necessarily represent those of their affiliated organizations, or those of the publisher, the editors and the reviewers. Any product that may be evaluated in this article, or claim that may be made by its manufacturer, is not guaranteed or endorsed by the publisher.

## References

[B1] Barker S. Dowe P. (2003). Paradoxes of multi-location. Analysis 63, 106–114. 10.1093/analys/63.2.106

[B2] Barker S. Dowe P. (2005). Endurance is paradoxical. Analysis 65, 69–74. 10.1093/analys/65.1.69

[B3] Baron S. Latham A. Miller K, Oh, J. (2022). Is endurantism the folk friendly view of persistence? Available online at: https://philpapers.org/rec/BARIET-4 (accessed August 16, 2022).

[B4] Baron S. Miller K. (2018). An Introduction to the Philosophy of Time. Cambridge: Polity Press.

[B5] Brower J. E. (2010). Aristotelian endurantism: a new solution to the problem of temporary intrinsics. Mind 119, 883–905. 10.1093/mind/fzq072

[B6] Buonomano D Rovelli C. (2022). Bridging the Neuroscience and Physics of Time. Time and Science

[B7] Callender C. (2017). What Makes Time Special. Oxford: OUP. 10.1093/oso/9780198797302.001.0001

[B8] Dainton B. (2011). “Time, passage and immediate experience,” in Oxford Handbook of Philosophy of Time, ed C. Callender (Oxford: OUP). 10.1093/oxfordhb/9780199298204.003.0013

[B9] Daniels P. (2014). Occupy wall: a mereological puzzle and the burdens of endurantism. Austral. J. Philos. 92, 91–101. 10.1080/00048402.2013.820764

[B10] Deng N. (2013). Our experience of passage on the B-theory. Erkenntnis 78, 713–726. 10.1007/s10670-013-9489-5

[B11] Deng N. (2019). “One thing after another: why the passage of time is not an illusion,” in The Illusions of Time: Philosophical and Psychological Essays on Timing and Time Perception, eds A. Bardon, V. Arstila, S. Power, and A. Vatakis (London: Palgrave Macmillan). 10.1007/978-3-030-22048-8_1

[B12] Eagle A. (2010). “Perdurance and location,” in Oxford Studies in Metaphysics, vol. 5, ed D. Zimmerman (Oxford), 53–94.

[B13] Effingham N. Robson J. (2007). A mereological challenge to endurantism. Austral. J. Philos. 85, 633–640. 10.1080/00048400701728541

[B14] Giberman D. (2017). Bent not broken: why exemplification simpliciter remains a problem for eternalist endurantism. Erkenntnis 82, 947–966. 10.1007/s10670-016-9852-4

[B15] Gruber R. P. Block R. A. Montemayor C. (2022). Physical time within human time. Front. Psychol. 13, 718505. 10.3389/fpsyg.2022.718505 35432085 PMC9005802

[B16] Hartle J. (2005). The physics of now. Am. J. Phys. 73, 101–109. 10.1119/1.1783900

[B17] Haslanger S. (1989). Endurance and temporary intrinsics. Analysis 49, 119–125. 10.1093/analys/49.3.119

[B18] Hoerl C. (2014). Do we (seem to) perceive passage? Philos. Explorat. 17, 188–202. 10.1080/13869795.2013.852615

[B19] Hoerl C. (2018). Experience and time: transparency and presence. Ergo 5, 127–151. 10.3998/ergo.12405314.0005.005

[B20] Ismael J. (2012). “Decision and the open future,” in The Future of the Philosophy of Time, ed A. Bardon (New York, NY: Routledge), 149–169.

[B21] Ismael J. (2017). “Passage, flow, and the logic of temporal perspectives,” in Time of Nature and the Nature of Time, eds C. Bouton and P. Huneman (Heidelberg: Springer Verlag), 10.1007/978-3-319-53725-2_2

[B22] Kriegel U. (2009). Temporally token-reflexive experiences. Can. J. Philos. 39, 585–617. 10.1353/cjp.0.0064

[B23] Latham A. J. Miller K. Norton J. (2019). Is our naïve theory of time dynamical? Synthese. 198, 4251–4271. 10.1007/s11229-019-02340-4

[B24] Latham A. J. Miller K. Norton J. (2020). Do the folk represent time as essentially dynamical? Inquiry. 1–32. 10.1080/0020174X.2020.1827027

[B25] Leininger L. (2021). Temporal B-coming: passage without presentness. Austral. J. Philos. 99, 130–147. 10.1080/00048402.2020.1744673

[B26] Merricks T. (1994). Endurance and indiscernibility. J. Philos. 91, 165–184. 10.2307/2940769

[B27] Merricks T. (1999). Persistence, parts, and presentism. Noûs 33, 421–438. 10.1111/0029-4624.00162

[B28] Miller K. (2004). Enduring special relativity. Southern J. Philos. 42, 349–370. 10.1111/j.2041-6962.2004.tb01937.x

[B29] Miller K. (2019). “Does it really seem as though time passes?” in The Illusions of Time: Philosophical and Psychological Essays on Timing and Time Perception, eds V. Artsila, A. Bardon, S. Power, and A. Vatakis (London: Palgrave McMillan), 17–33. 10.1007/978-3-030-22048-8_2

[B30] Miller K. Holcombe A. Latham A. J. (2020). Temporal phenomenology: phenomenological illusion versus cognitive error. Synthese 197, 751–771. 10.1007/s11229-018-1730-y

[B31] Peacocke C. (1999). Being Known. Oxford: Clarendon Press. 10.1093/0198238606.001.0001

[B32] Phillips I. (2014). Experience of and in time. Philos. Compass 9, 131–144. 10.1111/phc3.12107

[B33] Prosser S. (2007). Could we experience the passage of time? Ratio 20, 75–90. 10.1111/j.1467-9329.2007.00348.x

[B34] Prosser S. (2012). Why does time seem to pass? Philos. Phenomenol. Res. 85, 92–116. 10.1111/j.1933-1592.2010.00445.x

[B35] Prosser S. (2016). Experiencing Time. Oxford: Oxford University Press. 10.1093/acprof:oso/9780198748946.001.0001

[B36] Sattig T. (2019a). The flow of time in experience. Proc. Aristotelian Soc. 119, 275–293. 10.1093/arisoc/aoz014

[B37] Sattig T. (2019b). The sense of temporal flow: a higher-order account. Philos. Stud. 176, 3041–3059. 10.1007/s11098-018-1162-z

[B38] Sider T. (2001). Four Dimensionalism. Oxford: Clarendon Press. 10.1093/019924443X.001.0001

[B39] Wasserman R. (2016). Theories of persistence. Philos. Stud. 173, 243–250. 10.1007/s11098-015-0488-z

